# Clinical and Radiological Characteristics of Children and Adults With First-Attack Myelin Oligodendrocyte Glycoprotein Antibody Disease and Analysis of Risk Factors for Predicting the Severity at Disease Onset in Central China

**DOI:** 10.3389/fimmu.2021.752557

**Published:** 2021-12-15

**Authors:** Yanfei Li, Haojie Xie, Jinwei Zhang, Yongyan Zhou, Lijun Jing, Yaobing Yao, Ranran Duan, Yanjie Jia

**Affiliations:** Department of Neurology, The First Affiliated Hospital of Zhengzhou University, Zhengzhou, China

**Keywords:** myelin oligodendrocyte glycoprotein antibody disease, clinical and radiological characteristics, predictive factors, uric acid, homocysteine

## Abstract

**Objective:**

To analyze and compare different clinical, laboratory, and magnetic resonance imaging characteristics between pediatric and adult patients with first-attack myelin oligodendrocyte glycoprotein antibody disease (MOGAD) and to explore predictive factors for severity at disease onset.

**Methods:**

Patients diagnosed with MOGAD at the First Affiliated Hospital of Zhengzhou University from January 2013 to August 2021 were enrolled in this retrospective study. Age at disease onset, sex, comorbidities, laboratory tests, magnetic resonance imaging (MRI) characteristics, and Expanded Disability Status Scale (EDSS) scores were collected and analyzed. The association between risk factors and initial EDSS scores at disease onset was analyzed using logistic regression models and Spearman correlation analyses. A receiver-operating characteristic (ROC) curve analysis was used to evaluate the predictive ability of the uric acid and homocysteine (Hcy) levels for the severity of neurological dysfunction at the onset of MOGAD.

**Results:**

Sixty-seven patients (female, n=34; male, n=33) with first-attack MOGAD were included in this study. The mean age at onset was 26.43 ± 18.22 years (range: 3–79 years). Among patients <18 years of age, the most common presenting symptoms were loss of vision (36.0%), and nausea and vomiting (24.0%), and the most common disease spectrum was acute disseminated encephalomyelitis (ADEM) (40.0%). Among patients aged ≥18 years, the most common presenting symptoms were loss of vision (35.7%), paresthesia (33.3%), and paralysis (26.2%), and the most common disease spectrum was optic neuritis (35.7%). The most common lesions were cortical gray matter/paracortical white matter lesions in both pediatric and adult patients. Uric acid [odds ratio (OR)=1.014; 95% confidence interval (CI)=1.006–1.022; P=0.000] and serum Hcy (OR=1.125; 95% CI=1.017–1.246; P=0.023) levels were significantly associated with the severity of neurological dysfunction at disease onset. Uric acid levels (r=0.2583; P=0.035) and Hcy levels (r=0.3971; P=0.0009) were positively correlated with initial EDSS scores. The areas under the ROC curve were 0.7775 (95% CI= 0.6617‒0.8933; P<0.001) and 0.6767 (95% CI=0.5433‒0.8102, P=0.014) for uric acid and Hcy levels, respectively.

**Conclusion:**

The clinical phenotype of MOGAD varies in patients of different ages. The most common disease spectrum was ADEM in patients aged<18 years, while optic neuritis was commonly found in patients aged ≥18 years. The uric acid and Hcy levels are risk factors for the severity of neurological dysfunction at disease onset in patients with first-attack MOGAD.

## Introduction

Myelin oligodendrocyte glycoprotein antibody disease (MOGAD) is a rare autoimmune disorder characterized by antibodies against the myelin oligodendrocyte glycoprotein (MOG) and has a wide spectrum of presenting clinical phenotypes. The majority of patients with MOGAD present with acute disseminated encephalomyelitis (ADEM), transverse myelitis (TM), recurrent optic neuritis (ON), neuromyelitis optica spectrum disorders (NMOSD), and multiple sclerosis (MS) (1, 2). Approximately 40% of patients with NMOSD who are seronegative for the aquaporin-4 (AQP4) antibody have MOGAD (3). Unlike AQP4, which is an astrocytic protein, MOG is localized on the outer layer of myelin and oligodendrocytes, which may induce oligodendrocyte and myelin injuries that result in inflammatory demyelination in the central nervous system (CNS) (4).

The clinical and radiological findings of MOGAD vary. Previous studies have focused on the clinical and radiological features or treatment and prognosis of patients with MOGAD (5–7); studies reporting detailed laboratory test data are rare. Although many cases have been reported in recent years, Chinese studies regarding MOGAD include relatively small sample sizes. Studies identifying reliable and sensitive markers to predict the severity of neurological impairment in patients with NMOSD and MS have been reported (8, 9); however, few studies have reported predictive factors for the severity of MOGAD at disease onset.

Clinical, laboratory, and magnetic resonance imaging (MRI) characteristics were analyzed, and predictive factors for the severity at disease onset of MOGAD were identified in this study. We focused on the first-attack MOGAD patients to rule out possible effects of previous treatments (such as glucocorticoids, immunoglobulin, and immunosuppressants) on laboratory results.

This is the first study to investigate predictive factors for the severity of MOGAD at disease onset, and the results will be useful for the early assessment of the prognosis of patients with MOGAD, allowing for more individualized treatment plans.

## Materials and Methods

### Participants

Patients diagnosed with MOGAD at the First Affiliated Hospital of Zhengzhou University from January 2013 to August 2021 were retrospectively enrolled in this study. Patients who were seropositive for MOG antibodies on a live cell-based assay (CBA) with inflammatory attacks of the optic nerve, spinal cord, or brain were included (6).

**Figure 1:**
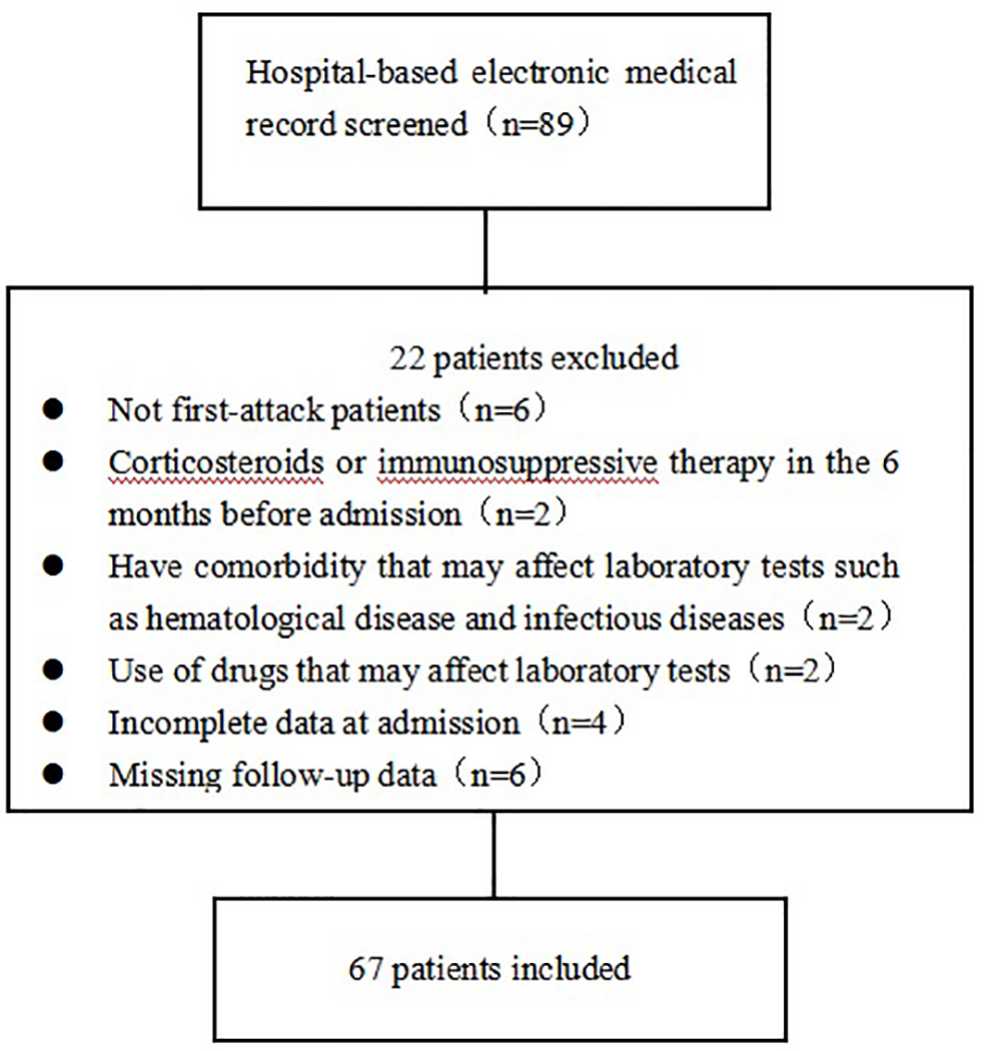
Overview of the patient selection process.

The exclusion criteria were a non-first-attack of MOGAD, the coexistence of other diseases that may affect the Expanded Disability Status Scale (EDSS) scores, the use of corticosteroids or immunosuppressive therapies in the six months prior to admission, the use of drugs that may affect laboratory tests (including lipid-lowering drugs, homocysteine-lowering drugs, or hepatic or renal protectants), and the presence of hematological, infectious, or other diseases that may affect the laboratory tests or cerebrospinal fluid (CSF) analysis. The detailed selection process is shown in **Figure 1**.

This study was approved by the Ethics Committee of First Affiliated Hospital of Zhengzhou University (2019-KY-018) and was conducted according to the principles of the Declaration of Helsinki. All participants or their guardians provided written informed consent for their participation in the study.

### Data Collection and Treatment

Age at onset, sex, comorbidities, clinical symptoms, treatments, laboratory tests [routine blood tests, liver function, renal function, lipids, thyroid hormones, erythrocyte sedimentation rate (ESR), C-reactive protein (CRP), homocysteine (Hcy), and antinuclear antibodies (ANAs)], CSF analysis (intracranial pressure, leukocyte count, and protein concentration), and imaging findings at admission were collected. The EDSS scores at admission, discharge, and follow-up were evaluated by an experienced neurologist. The clinical symptoms at admission were used to calculate the initial EDSS scores. Patients received different treatments according to their clinical symptoms and financial situation. Treatments including corticosteroids, immunoglobulins, and immunosuppressants (such as azathioprine, mycophenolate mofetil, and methotrexate) were recorded. Relapse was defined as new-onset or recurrent neurological symptoms lasting for at least 24 hours, resulting in an increase in the EDSS score of at least 0.5 points from the patient’s lowest score. Relapse events occurring within 28 days of one another were considered a single relapse (10). Follow-up data were obtained *via* annual clinic visits or telephone interviews. The last follow-up date was November 15, 2021.

Blood samples were collected from patients after overnight fasting at 7:00–8:00 am the next day after admission. Blood and CSF samples were obtained prior to the administration of any treatments. MOG antibodies were measured by CBA. When MOG antibody titers were more than 1:10, we considered the MOG status positive. The MOG antibody titers of participants varied from 1:10 to 1:000++. Titers more than 1:000 were described as 1:000+ or 1:1000++. The tests were conducted in accordance with the manufacturer’s protocols, and the examiners were blinded to the patients’ diagnoses and clinical symptoms.

### Imaging Data

MRI scans were performed using a 3.0T scanner (Philips Healthcare, Amsterdam, Netherlands). Sagittal T1-weighted images (T1WI), axial T1WI, T2-weighted images (T2WI), axial/sagittal fast fluid-attenuated inversion recovery (FLAIR) images, axial diffusion, apparent diffusion coefficient (ADC) mapped images, and contrast-enhanced axial, coronal, and sagittal T1WI images of the brain and sagittal T1WI, sagittal T2WI, axial T1WI, and axial T2WI images of the spine were analyzed. A gadolinium glutamine injection was used as the contrast agent. The locations of the lesions were recorded as deep white matter (WM), cortical gray matter/paracortical WM, periventricular WM, corpus callosum, basal ganglia, thalamus, midbrain, pons, medulla oblongata, cerebellum, cervical medulla, lumbar medulla, thoracic medulla, optic nerve, optic chiasma, and optic tract. All assessments were performed by two independent radiologists who were blinded to the patients’ clinical features.

### Grouping

The patients were divided into children (<18 years) and adults (≥18 years) based on the age at onset. The differences in clinical, laboratory, and radiological characteristics between children and adult patients were analyzed. The patients were then categorized based on initial EDSS score (≤3 or >3) to identify predictive factors for the severity of neurological dysfunction at disease onset (11).

### Statistical Analyses

Continuous data with a normal distribution are presented as mean ± standard deviation. Continuous data with a non-normal distribution are presented as median ± interquartile range. Categorical variables are expressed as frequency (percentage, %). The differences between the two groups were analyzed using the Student’s t-test and Wilcoxon test for normally and non-normally distributed data, respectively. Categorical data were compared using the chi-square test when comparing numbers ≥5 or Fisher’s exact test when comparing small numbers <5. A univariate logistic regression analysis was used to identify potential predictive factors for the severity of neurological dysfunction at the onset of MOGAD. A multivariate logistic regression analysis was used to determine the independent predictive factors for the severity of neurological dysfunction at disease onset. Variables with a significance level of P < 0.1 in the univariate logistic regression analysis were included in the basic model. Variables clinically believed to have an impact on the initial EDSS (including age at onset and sex) and factors that affect Hcy levels (including folic acid and vitamin B12) were included in the adjusted model to analyze the stability of the associations between uric acid levels, Hcy levels, and the severity of neurological dysfunction at disease onset. A correlation analysis was performed using the Spearman correlation analysis. Receiver-operating characteristic (ROC) curve analyses were used to evaluate the diagnostic value of the uric acid and Hcy levels for the severity of neurological dysfunction at the onset of MOGAD.

All statistical analyses were performed using SPSS (version 26.0; IBM, Armonk, NY, USA). Statistical significance was set at P < 0.05.

## Results

### Patient Demographics and Clinical Characteristics

Eighty-nine patients were diagnosed with MOGAD and were seropositive for MOG antibodies between January 2013 and August 2021 at First Affiliated Hospital of Zhengzhou University. Sixty-seven (females, n=34; males, n=33) of these patients met the inclusion criteria and were included in this study.

The mean patient age at onset was 26.43 ± 18.22 years (range: 3–79 years). A total of 25 patients were <18 years of age (mean age: 9.12 ± 3.71 years; 15 females), and 42 patients were ≥18 years of age (mean age: 36.74 ± 15.32 years; 19 females). Sex was not significantly different between the groups (**Table 1**).

**Table 1:** Patient demographics and comorbidities.

	All patients	Age<18	Age≥18	*P* value
	(n = 67)	(n = 25)	(n = 42)	
Age at onset	26.43 ± 18.22	9.12 ± 3.71	36.74 ± 15.32	0.000^*^
Sex, female	34 (50.7)	15 (60.0)	19 (45.2)	0.242
Smoking	8 (11.9)	0	8 (19.4)	0.020^*^
Drinking	4 (6.0)	0	4 (9.5)	0.289
Hypertension	3 (4.5)	0	3 (7.1)	0.288
Diabetes	1 (1.5)	0	1 (2.4)	1.000
Coronary heart disease	3 (4.5)	0	3 (7.1)	0.288
Cerebrovascular disease	0	0	0	1.000
Anxiety/depression	2 (3.0)	0	2 (4.8)	0.525
Malignancy	1 (1.5)	0	1 (2.4)	1.000
Trauma	1 (1.5)	1 (4.0)	0	0.373
Autoimmune diseases				
Sjogren syndrome	1 (1.5)	0	1 (2.4)	1.000
Thyroid disease	5 (7.5)	2 (8.0)	3 (7.1)	1.000

Data are presented as number (percentage) or mean ± standard deviation.

^*^P < 0.05.

The most common clinical symptoms were loss of vision (35.8%), paresthesia (28.4%), and paralysis (23.9%), and the main disease spectrums included ON (35.8%) and TM (26.9%). Among patients <18 years old, the most common presenting symptoms were visual loss (36.0%) and nausea/vomiting (24.0%), and the main disease spectrum was ADEM (40.0%). Among patients ≥18 years old, the most common presenting symptoms were loss of vision (35.7%), paresthesia (33.3%), and paralysis (26.2%), and the most common disease spectrum was ON (35.7%). The incidences of nausea and vomiting (P=0.009), post polar syndrome (P=0.009), and ADEM (P=0.000) were significantly higher in patients <18 years old than in patients ≥18 years old.

The initial, discharge, and follow-up EDSS scores were not significantly different between the two groups (P>0.05). Most patients (94.0%) received corticosteroids, while 14 (20.9%) received intravenous immunoglobulins, and 8 (11.9%) received immunosuppressants. The treatments were not significantly different between the groups (P>0.05).

The median follow-up period was 7 months (range: 3–42 months). A total of 14 (20.9%) patients had a relapse during the study period. The median time between the first and second episodes was 5.5 months (range: 2–26 months). There were no significant differences in follow-up times (P=0.109) or relapse rates (P=0.63) between the two groups (**Table 2**).

**Table 2:** Patient characteristics.

	All patients	Age<18	Age≥18	*P* value
	(n = 67)	(n = 25)	(n = 42)	
Symptoms at onset				
Visual loss	24 (35.8)	9 (36.0)	15 (35.7)	0.981
Paralysis	16 (23.9)	5 (20.0)	11 (26.2)	0.636
Paresthesia	19 (28.4)	5 (20.0)	14 (33.3)	0.242
Nausea/Vomiting	7 (10.4)	6 (24.0)	1 (2.4)	0.009^*^
Fever	10 (14.9)	5 (20.0)	5 (11.9)	0.368
Headache	10 (14.9)	2 (8.0)	8 (19.0)	0.300
Dizziness	7 (10.4)	1(4.0)	6 (14.3)	0.244
Seizures	12 (17.9)	5 (20.0)	7 (16.7)	0.731
Speech disorders	2 (3.0)	1 (4.0)	1 (2.4)	1.000
Unsteady gait	5 (7.5)	4 (16.0)	1 (2.4)	0.061
Disturbance of consciousness	2 (3.0)	1 (4.0)	1 (2.4)	1.000
Disease spectrum				
Optic neuritis	24 (35.8)	9 (36.0)	15 (35.7)	0.981
Transverse myelitis	18 (26.9)	9 (36.0)	9 (21.4)	0.193
Acute disseminated encephalomyelitis	11(16.4)	10 (40.0)	1 (2.4)	0.000^*^
Brainstem syndrome	11 (16.4)	4 (16.0)	7 (16.7)	1.000
Post polar syndrome	7 (10.4)	6 (24.0)	1 (2.4)	0.009^*^
Treatment				
Corticosteroid	63 (94.0)	25 (100)	38 (90.5)	0.112
Intravenous immunoglobulin	14 (20.9)	7 (28.0)	7 (16.7)	0.270
Immunosuppressant				
Azathioprine	4 (6.0)	0	4 (9.5)	0.289
Mycophenolate mofetil	3 (4.5)	1 (4.0)	2 (4.8)	1.000
Methotrexate	1 (1.4)	1 (4.0)	0	0.373
Initial EDSS	4 (2–6)	4 (3–4.75)	4 (2–6)	0.974
Discharge EDSS	2 (1–4)	1.5 (1–3)	2 (1–4)	0.351
Follow-up EDSS	1.5 (1–2)	1 (1–2)	1.5 (1–2)	0.340
Follow-up interval (months)	7 (5–15)	6 (3.5–8.5)	8 (5.375–16.75)	0.109
Relapse	14 (20.9)	6 (24.0)	8 (19.0)	0.630

Data are presented as mean ± standard deviation, number (percentage), or median (interquartile range).

EDSS, expanded disability status scale scores.

^*^P < 0.05.

### Laboratory Examinations

Patients ≥18 years old had higher total bilirubin (P=0.001), creatinine (P=0.000), intracranial pressure (P=0.048), and CSF protein levels (P=0.000) than patients <18 years old (**Table 3**). Among all patients, the median intracranial pressure during lumbar puncture was normal, while the median CSF leukocyte count was elevated. Eight patients (11.9%) had positive CSF oligoclonal bands (OBs). Three patients (4.5%) had anti-N-methyl-D-aspartate receptor (anti-NMDAR) antibodies during the first attack of MOGAD (two patients presented with loss of vision and one patient with paralysis). Among the three patients, the disease spectrum was ON for two patients and myelitis for the other. The follow-up durations of the three patients were 20 months, 8 months, and 7 months, respectively. None of the three patients met the diagnostic criteria for anti-NMDAR autoimmune encephalitis or relapsed during the study period.

**Table 3:** Laboratory data.

	All = patients	Age<18	Age≥18	*P* value
	(n = 67)	(n = 25)	(n = 42)	
Leukocyte counts (×10^9^/L)	9563 ± 5.84	11.64 ± 8.26	8.36 ± 3.30	0.068
Erythrocyte counts (×10^12^/L)	4.39 ± 0.49	4.48 ± 0.41	4.33 ± 0.52	0.233
Hemoglobin (g/L)	128 (118.1–142.9)	124 (119.85–135)	132 (118–144.35)	0.218
Lymphocyte count (×10^9^/L)	1.96 ± 0.99	2.14 ± 1.19	1.86 ± 0.86	0.268
Glucose (mmol/L)	4.93 ± 1.12	5.02 ± 1.20	4.88 ± 1.09	0.612
Total protein (g/L)	66.63 ± 5.99	68.81 ± 4.77	65.33 ± 6.31	0.013*
Total bilirubin (μmol/L)	8.00 ± 4.65	6.0 ± 2.19	9.19 ± 5.30	0.001^*^
Uric acid (μmol/L)	257.61 ± 84.72	246.60 ± 100.54	264.17 ± 74.28	0.452
Creatinine (μmol/L)	54.51 ± 16.44	41.96 ± 15.96	61.98 ± 11.53	0.000^*^
Total cholesterol (mmol/L)	3.79 ± 0.96	3.57 ± 0.45	3.92 ± 1.10	0.114
Triglycerides (mmol/L)	1.23 ± 0.82	1.21 ± 0.66	1.24 ± 0.91	0.892
High-density lipoprotein (mmol/L)	1.19 ± 0.32	1.17 ± 0.25	1.20 ± 0.36	0.687
Low-density lipoprotein (mmol/L)	2.29 ± 0.72	2.07 ± 0.53	2.42 ± 0.80	0.055
ESR (mm/h)	10 (6–17)	11 (6–16.5)	8.65 (5.875–18.25)	0.484
CRP (mg/L)	1.82 (0.5–4.7)	1 (0.425–5.23)	2.705 (0.975–4.385)	0.248
FT3 (pmol/L)	4.88 ± 0.97	5.04 ± 1.15	4.78 ± 0.85	0.323
FT4 (pmol/L)	13.06 ± 2.86	13.59 ± 2.73	12.74 ± 2.92	0.243
TSH (pmol/L)	2.02 ± 1.35	1.68 ± 1.11	2.22 ± 1.45	0.114
Homocysteine levels (μmol/L)	14.74 ± 6.30	15.31 ± 6.56	14.40 ± 6.19	0.574
Folic acid (ng/mL)	7.59 ± 4.02	6.60 ± 3.21	8.19 ± 4.36	0.119
Vitamin B12 (pg/mL)	591.76 ± 397.33	614.66 ± 367.69	578.14 ± 417.72	0.719
ANAs positive	17 (25.4)	3 (12.0)	14 (33.3)	0.081
Thyroid peroxidase/Thyroglobulin antibodies positive	7 (10.4)	3 (12.0)	4 (9.5)	1.000
Intracranial pressure (mmH2O)	160 (135–180)	150 (135–170)	165 (140–200)	0.048^*^
CSF leukocyte counts (×10^6^/L)	12 (4–32)	15 (4.5–42)	11 (3.75–30.5)	0.266
CSF protein concentration (mg/L)	309.8 (233.2–388.9)	238 (183.55–306)	361.15 (294.525–446.875)	0.000^*^
Positive oligoclonal band	8 (11.9)	5 (20.0)	3 (7.1)	0.138
CSF anti-NMDAR(+)	3 (.4.5)	1 (4.0)	2 (4.8)	1.000
Serum AQP4-IgG(+)	0	0	0	–

Data are presented as mean ± standard deviation, number (percentage), or median (interquartile range).

NLR, neutrophil-to-lymphocyte ratio; ESR, erythrocyte sedimentation rate; CRP, C-reactive protein; FT3, free triiodothyronine; FT4, free thyroxine; TSH, thyroid-stimulating hormones; ANAs, antinuclear antibodies; CSF, cerebrospinal fluid; anti-NMDAR, anti-N-methyl-D-aspartate receptor; AQP4-IgG, aquaporin-4 immunoglobulins G.

^*^P < 0.05.

### Radiological Characteristics

At disease onset, nine patients (13.4%) had normal brain or spinal cord MRIs, and then six patients presented abnormal MRI findings in subsequent disease processes. MOGAD lesions were identified in the optic nerve, cortical gray matter/paracortical WM, deep WM, periventricular WM, corpus callosum, basal ganglia, brainstem, cerebellum, and spinal cord. Overall, 41.8% of patients had lesions in the cortical gray matter/paracortical WM. The cortical gray matter/paracortical WM was the most common location for MOGAD lesions in both patients <18 years old and ≥18 years old. The cervical medulla was more involved than thoracic and lumbar lesions of the spinal cord (**Table 4**).

**Table 4:** Patients’ radiological characteristics.

	All patients	Age<18	Age≥18	*P* value
	(n = 67)	(n = 25)	(n = 42)	
Brain MRI				
Optic nerve	9 (13.4)	4 (160)	5 (11.9)	0.718
Cortical gray matter/paracortical WM	28 (41.8)	10 (40.0)	18 (42.9)	0819
Deep WM	9 (13.4)	3 (12.0)	6 (14.3)	1.000
Periventricular WM	15 (22.4)	6 (24)	9 (21.4)	0.807
Corpus callosum	2 (3.0)	1 (4.0)	1 (2.4)	1.000
Basal ganglia	7 (10.4)	4 (16.0)	3 (7.1)	0.411
Thalamus	10 (14.9)	3 (12.0)	7 (16.7)	0.732
Midbrain	5 (7.5)	2 (8.0)	3 (7.1)	1.000
Pons	14 (20.9)	6 (24.0)	8 (19.0)	0.630
Medulla oblongata	8 (11.9)	6 (24.0)	2 (4.8)	0.045^*^
Cerebellum	8 (11.9)	4 (16.0)	4 (9.5)	0.459
Spine MRI				
Cervical medulla	17 (25.4)	7 (28.0)	10 (23.8)	0.703
Thoracic medulla	16 (23.9)	6 (24.0)	10 (23.8)	0.986
Lumbar medulla	2 (3.0)	0	2 (4.8)	0.525

Data are presented as number (percentage).

MRI, magnetic resonance imaging; WM, white matter.

^*^P < 0.05.

### Predictive Factors for Disease Severity

There were no significant differences in age at onset, sex, erythrocyte count, hemoglobin, lymphocyte count, glucose, total protein, total bilirubin, creatinine, total cholesterol, triglycerides, high-density lipoprotein, ESR, CRP, free triiodothyronine (FT3), free thyroxine (FT4), thyroid-stimulating hormones (TSH), folic acid, vitamin B12, intracranial pressure, and CSF leukocyte count and protein concentration between the patients with an EDSS score >3 and those with an EDSS score ≤ 3 (**Table 5**). The proportions of positive ANAs (P=0.530) and positive thyroid peroxidase or thyroglobulin (P=0.454) antibodies were similar between the two groups. Patients with an EDSS score ≤3 had lower uric acid (P=0.000), low-density lipoprotein (P=0.013), and Hcy levels (P=0.015) than patients with an EDSS score >3.

**Table 5:** Laboratory data according to initial Expanded Disability Status Scale.

	EDSS ≤3	EDSS>3	*P* value
	(n = 28)	(n = 39)	
Age at onset, years	24.43 ± 16.52	27.87 ± 19.43	0.450
Sex, female	14 (50.0)	20 (51.3)	0.918
Leukocyte count (×10^9^/L)	10.91± 7.83	8.64 ± 3.68	0.118
Erythrocyte count (×10^12^/L)	4.39 ± 0.59	4.39 ± 0.41	0.950
Hemoglobin (g/L)	126.85 (116.5–145.775)	128 (122–142)	0.652
Lymphocyte count (×10^9^/L)	1.82 ± 0.73	2.07 ± 1.14	0.325
Glucose (mmol/L)	4.73 ± 1.00	5.08 ± 1.20	0.219
Total protein (g/L)	66.82 ± 6.30	66.49 ± 5.84	0.830
Total bilirubin (μmol/L)	7.41 ± 2.86	8.42 ± 5.59	0.335
Uric acid (μmol/L)	209.96 ± 77.03	291.82 ± 73.32	0.000^*^
Creatinine (μmol/L)	54.93 ± 16.62	54.21 ± 16.53	0.861
Total cholesterol (mmol/L)	3.61 ± 0.75	3.92 ± 1.08	0.188
Triglycerides (mmol/L)	1.13 ± 0.87	1.30 ± 0.78	0.404
High-density lipoprotein (mmol/L)	1.18 ± 0.33	1.19 ± 0.31	0.955
Low-density lipoprotein (mmol/L)	2.05 ± 0.50	2.46 ± 0.81	0.013^*^
ESR (mm/h)	8 (5.1–16.5)	10 (7–18)	0.184
CRP (mg/L)	1.585 (0.625–3.7725)	2.31 (0.5–5.14)	0.736
FT3 (pmol/L)	4.82 ± 0.83	4.92 ± 1.06	0.670
FT4 (pmol/L)	12.93 ± 2.60	13.15 ± 3.06	0.757
TSH (pmol/L)	1.85 ± 1.33	2.14 ± 1.37	0.389
Homocysteine levels (μmol/L)	12.56 ± 4.37	16.31 ± 7.02	0.015^*^
Folic acid (ng/mL)	7.69 ± 3.54	7.52 ± 4.38	0.867
Vitamin B12 (pg/mL)	487.83 ± 181.10	666.38 ± 486.52	0.069
ANAs positive	6 (15.4)	11 (28.2)	0.530
Thyroid peroxidase/Thyroglobulin antibodies positive	2 (5.1)	5 (12.8)	0.454
Intracranial pressure (mmH2O)	160 (126.25–180)	160 (140–175)	0.592
CSF leukocyte counts (×10^6^/L)	10 (4–27.75)	14 (4–40)	0.511
CSF protein concentration (mg/L)	309.6 (226.75–369.175)	309.8 (238–440.2)	0.457

Data are presented as mean ± standard deviation, number (percentage), or median (interquartile range).

NLR, neutrophil-to-lymphocyte ratio; ESR, erythrocyte sedimentation rate; CRP, C-reactive protein; FT3, free triiodothyronine; FT4, free thyroxine; TSH, thyroid-stimulating hormones; ANAs, antinuclear antibodies; CSF, cerebrospinal fluid; EDSS, Expanded Disability Status Scale.

^*^P < 0.05.

Uric acid (odds ratio (OR)=1.014; 95% confidence interval (CI)=1.006–1.022; P=0.000) and serum Hcy levels (OR=1.125; 95% CI=1.017–1.246; P=0.023) were significantly correlated with the initial EDSS score (**Table 6**). In the basic model of multivariate logistic regression analysis, uric acid (OR=1.014; 95% CI=1.004–1.023; P=0.003) and Hcy levels (OR=1.125; 95% CI=1.002–1.262; P=0.045) were related with the severity of neurological dysfunction at the onset of MOGAD. In the adjusted model, uric acid (OR=1.019; 95% CI=1.007–1.031; P=0.002) and Hcy levels (OR=1.198; 95% CI=1.033–1.390; P=0.017) remained significantly correlated with the severity of neurological dysfunction at the onset of MOGAD (**Table 7**).

**Table 6:** Univariate logistic regression analysis of potential predictive factors for the severity at the onset of myelin oligodendrocyte glycoprotein antibody disease.

Variables	Univariate analysis	*P* value
	OR (95% CI)	
Age at onset	1.011 (0.983–1.039)	0.444
Sex	0.950 (0.360–2.509)	0.918
Leukocyte count	0.920 (0.819–1.034)	0.163
Erythrocyte count	0.966 (0.353–2.646)	0.946
Hemoglobin	1.005 (0.984–1.027)	0.638
Lymphocyte count	1.301 (0.772–2.194)	0.323
Glucose	1.338 (0.840–2.130)	0.220
Total protein	0.991 (0.913–1.075)	0.826
Total bilirubin	1.053 (0.938–1.181)	0.382
Uric acid	1.014 (1.006–1.022)	0.000^*^
Creatinine	0.997 (0.968–1.027)	0.858
Total cholesterol	1.437 (0.835–2.473)	0.191
Triglycerides	1.310 (0.698-2.456)	0.401
High-density lipoprotein	1.045 (0.226–4.830)	0.955
Low-density lipoprotein	2.496 (1.108–5.623)	0.027^*^
ESR	1.029 (0.983–1.077)	0.216
CRP	0.978 (0.932–1.027)	0.372
FT3	1.120 (0.671–1.867)	0.665
FT4	1.028 (0.866–1.221)	0.753
TSH	1.183 (0.810–1.728)	0.385
Homocysteine levels	1.125 (1.017–1.246)	0.023^*^
Folic acid	0.989 (0.876–1.117)	0.864
Vitamin B12	1.001 (1.000–1.003)	0.086
ANAs positive	0.694 (0.222–2.172)	0.531
Thyroid peroxidase/Thyroglobulin antibodies positive	0.523 (0.094–2.914)	0.460
Intracranial pressure	1.006 (0.992–1.020)	0.400
CSF leukocyte count	1.003 (0.990–1.015)	0.670
CSF protein concentration	1.002 (0.999–1.006)	0.223

NLR, neutrophil-to-lymphocyte ratio; ESR, erythrocyte sedimentation rate; CRP, C-reactive protein; FT3, free triiodothyronine; FT4, free thyroxine; TSH, thyroid stimulating hormones; ANAs, antinuclear antibodies; CSF, cerebrospinal fluid; OR, odds ratio; CI, confidence interval.

^*^P < 0.05.

**Table 7:** Multivariate logistic regression analysis of predictive factors for the severity at the onset of myelin oligodendrocyte glycoprotein antibody disease.

Variables	Multivariate analysis			
	^a^Basic Model		^b^Adjusted model	
	OR (95% CI)	*P* value	OR (95% CI)	*P* value
Age			1.014 (0.976–1.054)	0.475
Sex, female			0.262 (0.051–1.339)	0.108
Uric acid	1.014 (1.004–1.023)	0.003^*^	1.019 (1.007–1.031)	0.002^*^
Low-density lipoprotein	2.938 (1.068–8.088)	0.037^*^	2.298 (0.818–6.456)	0.114
Homocysteine levels	1.125 (1.002–1.262)	0.045^*^	1.198 (1.033–1.390)	0.017^*^
Folic acid			1.003 (0.829–1.214)	0.973
Vitamin B12	1.002 (0.999–1.005)	0.140	1.002 (0.999–1.005)	0.174

a Basic Model: Variables with P < 0.1 in the univariate logistic regression analysis were included in the multivariate model.

b Adjusted model: Variables with P < 0.1 in the univariate logistic regression analysis or variables clinically believed to have an impact on the initial Expanded Disability Status Scale (including age at onset and sex) and factors that affect Hcy levels (including folic acid and vitamin B12) were included in the adjusted model.

*P < 0.05.

CI, confidence interval; OR, odds ratio.

**Figure 2:**
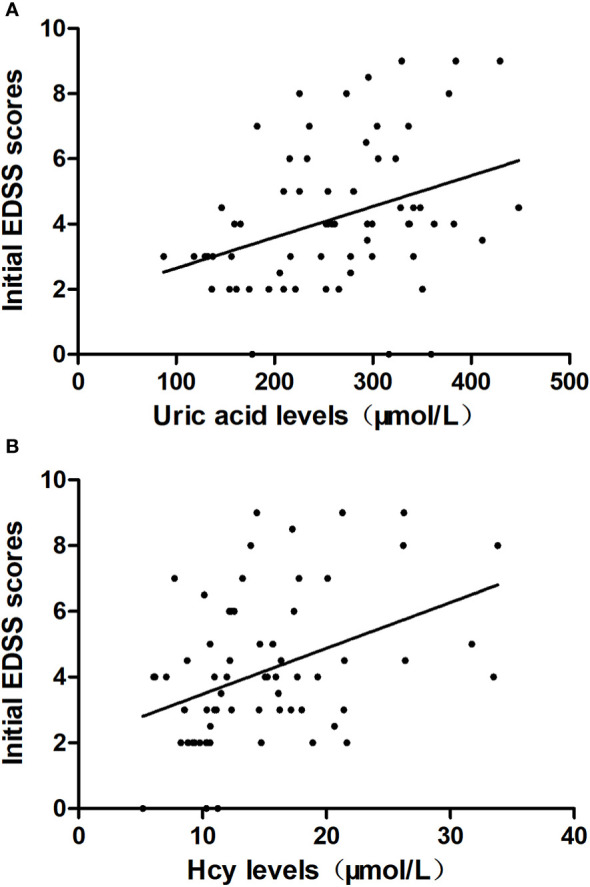
The correlation between uric acid levels, homocysteine (Hcy) levels and the initial Expanded Disability Status Scale (EDSS) scores. **(A)** The correlation between uric acid levels and the initial EDSS scores is shown. **(B)** The correlation between Hcy levels and the initial EDSS scores is shown.

Uric acid levels (r=0.3905; P=0.0011) and Hcy levels (r=0.3971; P=0.0009) were found to be positively correlated with the initial EDSS scores (**Figure 2**).

**Figure 3:**
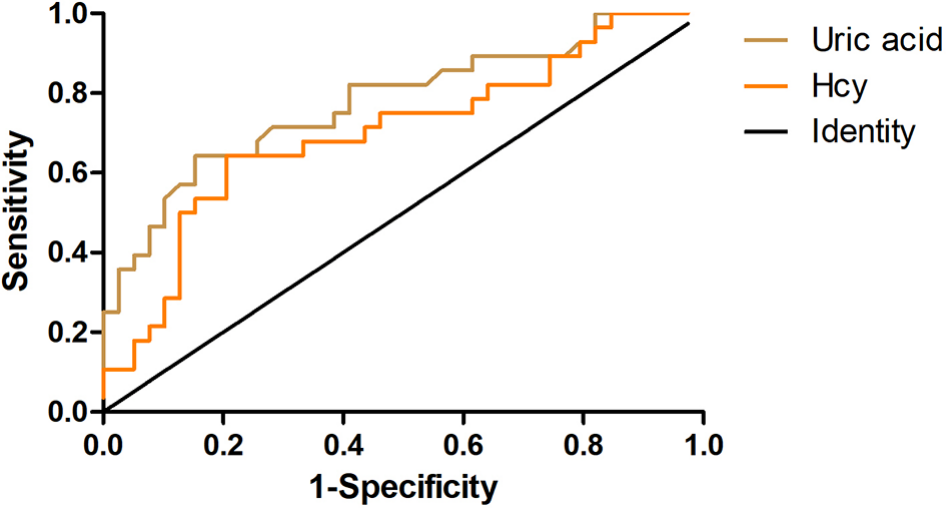
The receiver operating characteristic (ROC) curves. The ROC curves show the predictive abilities of uric acid (light brown) and serum homocysteine (orange) for the severity of neurological dysfunction at the onset of myelin oligodendrocyte glycoprotein antibody disease (MOGAD).

ROC curve analysis was used to evaluate the predictive value of uric acid and Hcy for predicting the severity of neurological impairment at the onset of MOG. The areas under the ROC curve were 0.7775 (95% CI= 0.6617‒0.8933; P<0.001) for uric acid levels and 0.6767 (95% CI=0.5433‒0.8102, P=0.014) for Hcy levels. At a uric acid cut-off value of 223 μmol/L, the sensitivity for predicting an initial EDSS>3 was 64.29% and the specificity was 84.62%. At an Hcy cut-off value of 11.37 μmol/L, the sensitivity for predicting an initial EDSS>3 was 64.29% and the specificity was 79.49% (**Figure 3**).

## Discussion

MOGAD is an antibody-mediated inflammatory demyelinating disease of the CNS with a monophasic or relapsing course of neurological dysfunction. It presents as various phenotypes, such as ADEM, TM, recurrent ON, and cortical encephalitis (12, 13). In this study, we carried out a retrospective analysis to explore and compare different clinical, laboratory, and magnetic resonance imaging characteristics between children and adult patients with first-attack MOGAD, and explored risk factors for predicting the severity at disease onset of MOGAD. We found that the clinical phenotype of MOGAD varies in patients of different ages. The most common disease spectrum was ADEM in patients aged <18 years, while ON was commonly found in patients aged ≥18 years. The most common lesions were cortical gray matter/paracortical WM lesions in both pediatric and adult patients. Uric acid and Hcy levels were associated with the severity of neurological dysfunction at the disease onset of MOGAD. To the best of our knowledge, this is the first study to explore risk factors for predicting the severity of neurological dysfunction at the onset of MOGAD in a real-world setting. To eliminate the effects of previous treatments (such as glucocorticoids, immunoglobulin, and immunosuppressants) on laboratory indicators, we focused on the first-attack NMOSD patients in the study.

Among 67 patients with first-attack MOGAD, the age of onset ranged from 3 to 79 years with an average age of 26.43 years, which was consistent with previous reports (14, 15). Prior evidence suggests that the female-to-male ratio among NMOSD patients is about 8:1 for AQP4-seropositive patients and 2:1 for AQP4-seronegative patients (16). Unlike the higher proportion of affected females seen in NMOSD, we found no sex differences between the affected males and females in patients with MOGAD, with a female-to-male ratio of 1.03:1. Similarly, there was no sex difference in either children or adult groups.

Loss of vision was the most common symptom in both children and adults in this study, and the most common disease spectrum was ADEM in children and ON in adults. A previous study reported that among patients <14 years of age with MOGAD, ADEM was the most frequent initial clinical symptom follow/ed by ON (17). In the study, we found that the incidence of nausea/vomiting and post polar syndrome in children was significantly higher than that in adults. However, when compared with children with AQP4-IgG positive NMOSD, patients with MOGAD tend to be less likely to present with post polar syndrome, but more likely to present with ADEM (18).

A previous study showed that 33.8–54% of children experience clinical relapses (19). Another report indicated that the relapse rate of MOG-Ab-positive patients aged ≥18 years were 44.8% and 61.8% after 2 and 5 years, respectively (20). Our study found that 24% of children patients experienced relapse, and adult patients showed a relapse rate of 19%, which was lower than previously-reported rates. This difference may be due to the different mean follow-up intervals of the studies.

Most patients with MOGAD present normal intracranial pressure and CSF protein concentration. Median CSF leukocyte counts of MOGAD were slightly elevated in our study. A previous study indicated that positive OBs were more commonly found in children and occurred in 6–17% of MOGAD patients. In the present study, the incidence of OBs was 11.9%, consistent with a previous study (21). A cohort study enrolled 42 patients with MOGAD and 491 patients with NMOSD and found that 11.9% patients with MOGAD and 0.6% patients with NMOSD had overlapping anti-NMDAR encephalitis (22).Three patients (4.4%) with MOGAD were found to have anti-NMDAR antibodies at first attack in our study. A previous study found that patients with MOGAD with positive anti-NMDAR antibodies had a higher relapse compared with patients with anti-NMDAR encephalitis (17). We speculate the possible reason may be MOGAD causes oligodendrocyte damage and primary demyelination. N-acetyl aspartate (NAA) is mainly catabolised in oligodendrocytes. Defective NAA metabolism in oligodendrocytes may lead to increased NAA, which is a sign of acute neuronal damage (23). In the study, during follow-up, none of the three patients relapsed. Longer follow-up periods are required to further investigate the relapse rate in patients with MOGAD with anti-NMDAR antibodies.

Our study found that the most common lesions in children and adults were cortical gray matter/paracortical WM, which were present in 41.8% of all patients with MOGAD. Previous reports showed that in comparison with AQP4-positive NMOSD patients, MOGAD patients are more likely to have cortical gray matter/paracortical WM region involvement. Salama et al. reported that cortical gray matter/paracortical WM lesions on brain MRI might help distinguish MOGAD from AQP4-positive NMOSD (6). Thus, we speculated that cortical gray matter/paracortical WM might act as a potential imaging marker for MOGAD. Thalamic and pontine lesions have also been reported as more common in patients with MOGAD than in patients with AQP4-positive NMOSD (20). Bilateral thalamic lesions at onset have been reported in approximately 60% of children with MOGAD (15). We found that brainstem involvement, especially of the pons and medulla oblongata, was commonly found in children, whereas thalamic lesions were less common compared with a previous report (15). It has been reported that >50% of patients with MOGAD have hyperintense lesions in the spinal cord (especially in the cervical or thoracic regions) on T2 images (14). In the study, the incidence of spinal cord lesions was 52% in children and 52.4% in adults, respectively, with cervical or thoracic segments predominantly involved.

In this study, patients with an initial EDSS score ≤3 had lower levels of uric acid, low-density lipoproteins, and Hcy than patients with an initial EDSS score >3. Uric acid and Hcy levels were correlated with the severity of neurological dysfunction at the onset of MOGAD and with the initial EDSS score. The optimal cut-off values of uric acid and Hcy levels for predicting the severity of neurological dysfunction at the onset of MOGAD were 223μmol/L and 11.37 μmol/L, respectively.

Uric acid is a natural product of the adenine nucleotide metabolic pathway, and its role in the CNS remains unclear. Previous studies have reported that uric acid is a strong free radical scavenger and antioxidant, while other studies have shown that uric acid reflects the production of free radicals by xanthine oxidase and is related to the glutamate-mediated excitotoxicity in neurological patients, which reflects the rate of adenosine triphosphate (ATP) catabolism (24, 25). In the attempt to find out reliable diagnostic or prognostic biomarkers, uric acid in biological fluids (plasma/serum, CSF, and urine) was measured in patients with MS or NMOSD. The uric acid levels in the biological fluids of patients with MS or NMOSD remain controversial. Some previous studies observed decreased uric acid levels in both serum/CSF of MS and NMOSD patients. Researchers have speculated that MS/NMOSD patients with low uric acid levels were unable to inhibit free radical toxicity and inflammation occurring in diseases. To deplete the excessively produced free radicals, the consumption of uric acid increases with lower uric acid levels as a result (26, 27). Contrarily, other scientists have observed different results. Amorini et al. demonstrated that uric acid levels were significantly higher in both the CSF and serum of MS patients in comparison with control groups, leading to the hypothesis that uric acid does not act as an antioxidant but indicates ongoing accelerated purine catabolism, possibly secondary to energy imbalance in MS (28). Another study reported significantly increased uric acid levels in the CSF and mildly increased uric acid levels in the serum of patients with NMOSD during relapse compared to control patients. Patients with an EDSS score >3.5 were reported to have higher uric acid levels in the CSF than patients with an EDSS score ≤ 3.5 (29). In the present study, we demonstrated that uric acid levels were related to the severity of MOGAD. Further studies are needed to identify the possible mechanisms of uric acid in MOGAD.

Serum Hcy levels were found to be associated with the prognosis of MS and NMOSD (30, 31). Elevated Hcy levels could cause oxidative stress, mitochondrial dysfunction, myelin sheath degeneration, and apoptosis (32–34). In our previous study, we found that Hcy was an independent predictor of relapse and poor prognosis in first-attack NMOSD patients (31). In the present study, we found that serum Hcy levels were higher in patients with EDSS >3 than in patients with EDSS ≤3 and positively correlated with the severity of neurological dysfunction at onset of MOGAD.

This study had some limitations. First, the sample size was relatively small, and the patients were from a single center. Second, the follow-up period was relatively short. Finally, the antibody titer results of some patients were not recorded. Therefore, the results of this study require further validation in larger multicenter studies with longer follow-up periods.

In conclusion, the results of this study suggest that MOGAD presents differently in patients of different ages. Uric acid and Hcy levels may be useful predictors of the severity of MOGAD at onset during the first attack, and elevated uric acid and Hcy levels are associated with severe neurological disabilities. These results indicate that more aggressive therapies should be administered when these predictors are observed. Further studies are needed to validate these conclusions.

## Data Availability Statement

The raw data supporting the conclusions of this article will be made available by the authors, without undue reservation.

## Ethics Statement

The studies involving human participants were reviewed and approved by the Ethics Committee of First Affiliated Hospital of Zhengzhou University. Written informed consent to participate in this study was provided by the participants’ legal guardian/next of kin.

## Author Contributions

YL: Methodology, Formal analysis, Data curation, Writing—original draft, Writing—review and editing. HX: Investigation, Writing—review and editing. JZ: Investigation, Writing—review and editing. YZ: Methodology, Investigation, Writing—review and editing. LJ: Data curation. YY: Formal analysis. RD: Formal analysis. YJ: Conceptualization, Methodology, Supervision, Funding acquisition. The first draft of the manuscript was written by YL and all authors commented on previous versions of the manuscript. All authors contributed to the article and approved the submitted version.

## Conflict of Interest

The authors declare that the research was conducted in the absence of any commercial or financial relationships that could be construed as a potential conflict of interest.

## Publisher’s Note

All claims expressed in this article are solely those of the authors and do not necessarily represent those of their affiliated organizations, or those of the publisher, the editors and the reviewers. Any product that may be evaluated in this article, or claim that may be made by its manufacturer, is not guaranteed or endorsed by the publisher.

## References

[1] Hegen H Reindl M. Recent Developments in MOG-IgG Associated Neurological Disorders. Ther Adv Neurol Disord (2020) 13:1756286420945135. doi: 10.1177/1756286420945135 33029200 PMC7521831

[2] Ramanathan S Dale RC Brilot F. Anti-MOG Antibody: The History, Clinical Phenotype, and Pathogenicity of a Serum Biomarker for Demyelination. Autoimmun Rev (2016) 15(4):307–24. doi: 10.1016/j.autrev.2015.12.004 26708342

[3] Hamid SHM Whittam D Mutch K Linaker S Solomon T Das K. What Proportion of AQP4-IgG-Negative NMO Spectrum Disorder Patients Are MOG-IgG Positive? A Cross Sectional Study of 132 Patients. J Neurol (2017) 264(10):2088–94. doi: 10.1007/s00415-017-8596-7 PMC5617862 28840314

[4] Hennes EM Baumann M Lechner C Rostásy K. MOG Spectrum Disorders and Role of MOG-Antibodies in Clinical Practice. Neuropediatrics (2018) 49(1):3–11. doi: 10.1055/s-0037-1604404 28859212

[5] Denève M Biotti D Patsoura S Ferrier M Meluchova Z Mahieu L. MRI Features of Demyelinating Disease Associated With Anti-MOG Antibodies in Adults. J Neuroradiol (2019) 46(5):312–8. doi: 10.1016/j.neurad.2019.06.001 31228536

[6] Salama S Khan M Shanechi A Levy M Izbudak I. MRI Differences Between MOG Antibody Disease and AQP4 NMOSD. Mult Scler (2020) 26(14):1854–65. doi: 10.1177/1352458519893093 PMC7363520 31937191

[7] Chen JJ Bhatti MT. Clinical Phenotype, Radiological Features, and Treatment of Myelin Oligodendrocyte Glycoprotein-Immunoglobulin G (MOG-IgG) Optic Neuritis. Curr Opin Neurol (2020) 33(1):47–54. doi: 10.1097/WCO.0000000000000766 31743235

[8] He Q Li L Li Y Lu Y Wu K Zhang R. Free Thyroxine Level Is Associated With Both Relapse Rate and Poor Neurofunction in First-Attack Neuromyelitis Optica Spectrum Disorder (NMOSD) Patients. BMC Neurol (2019) 19(1):329. doi: 10.1186/s12883-019-1560-7 31852443 PMC6921452

[9] Shugaiv E Tüzün E Kürtüncü M Kıyat-Atamer A Çoban A Akman-Demir G. Uveitis as a Prognostic Factor in Multiple Sclerosis. Mult Scler (2015) 21(1):105–7. doi: 10.1177/1352458514539782 24948689

[10] Wu K Wen L Duan R Li Y Yao Y Jing L. Triglyceride Level Is an Independent Risk Factor in First-Attacked Neuromyelitis Optica Spectrum Disorders Patients. Front Neurol (2019) 10:1230. doi: 10.3389/fneur.2019.01230 31824407 PMC6881454

[11] Zhou Y Xie H Zhao Y Zhang J Li Y Duan R. Neutrophil- to–Lymphocyte Ratio on Admission Is an Independent Risk Factor for the Severity of Neurological Impairment at Disease Onset in Patients With a First Episode of Neuromyelitis Optica Spectrum Disorder. Neuropsychiatr Dis Treat (2021) 17:1493–503. doi: 10.2147/NDT.S311942 PMC8140946 34040376

[12] Jarius S Paul F Aktas O Asgari N Dale RC de Seze J. MOG Encephalomyelitis: International Recommendations on Diagnosis and Antibody Testing. J Neuroinflamm (2018) 15(1):134. doi: 10.1186/s12974-018-1144-2 PMC5932838 29724224

[13] Ambrosius W Michalak S Kozubski W Kalinowska A. Myelin Oligodendrocyte Glycoprotein Antibody-Associated Disease: Current Insights Into the Disease Pathophysiology, Diagnosis and Management. Int J Mol Sci (2020) 22(1):100. doi: 10.3390/ijms22010100 PMC7795410 33374173

[14] Mariotto S Ferrari S Monaco S Benedetti MD Schanda K Alberti D. Clinical Spectrum and IgG Subclass Analysis of Anti-Myelin Oligodendrocyte Glycoprotein Antibody-Associated Syndromes: A Multicenter Study. J Neurol (2017) 264(12):2420–30. doi: 10.1007/s00415-017-8635-4 PMC5688213 29063242

[15] Cobo-Calvo Á Ruiz A D’Indy H Poulat AL Carneiro M Philippe N. MOG Antibody-Related Disorders: Common Features and Uncommon Presentations. J Neurol (2017) 264(9):1945–55. doi: 10.1007/s00415-017-8583-z 28770374

[16] Quek AM McKeon A Lennon VA Mandrekar JN Iorio R Jiao Y. Effects of Age and Sex on Aquaporin-4 Autoimmunity. Arch Neurol (2012) 69(8):1039–43. doi: 10.1001/archneurol.2012.249 PMC3746965 22507888

[17] Mao L Yang L Kessi M He F Zhang C Wu L. Myelin Oligodendrocyte Glycoprotein (MOG) Antibody Diseases in Children in Central South China: Clinical Features, Treatments, Influencing Factors, and Outcomes. Front Neurol (2019) 10:868. doi: 10.3389/fneur.2019.00868 31440204 PMC6694759

[18] Hacohen Y Mankad K Chong WK Barkhof F Vincent A Lim M. Diagnostic Algorithm for Relapsing Acquired Demyelinating Syndromes in Children. Neurology (2017) 89(3):269–78. doi: 10.1212/WNL.0000000000004117 28615429

[19] Jarius S Ruprecht K Kleiter I Borisow N Asgari N Pitarokoili K. MOG-IgG in NMO and Related Disorders: A Multicenter Study of 50 Patients. Part 1: Frequency, Syndrome Specificity, Influence of Disease Activity, Long-Term Course, Association With AQP4-IgG, and Origin. J Neuroinflamm (2016) 13(1):279. doi: 10.1186/s12974-016-0717-1 PMC5084340 27788675

[20] Cobo-Calvo A Ruiz A Maillart E Audoin B Zephir H Bourre B. Clinical Spectrum and Prognostic Value of CNS MOG Autoimmunity in Adults: The MOGADOR Study. Neurology (2018) 90(21):e1858–69. doi: 10.1212/WNL.0000000000005560 29695592

[21] Wynford-Thomas R Jacob A Tomassini V. Neurological Update: MOG Antibody Disease. J Neurol (2019) 266(5):1280–6. doi: 10.1007/s00415-018-9122-2 PMC6469662 30569382

[22] Fan S Xu Y Ren H Guan H Feng F Gao X. Comparison of Myelin Oligodendrocyte Glycoprotein (MOG)-Antibody Disease and AQP4-IgG-Positive Neuromyelitis Optica Spectrum Disorder (NMOSD) When They Co-Exist With Anti-NMDA (N-Methyl-D-Aspartate) Receptor Encephalitis. Mult Scler Relat Disord (2018) 20:144–52. doi: 10.1016/j.msard.2018.01.007 29414288

[23] Tortorella C Ruggieri M Di Monte E Ceci E Iaffaldano P Direnzo V. Serum and CSF N-Acetyl Aspartate Levels Differ in Multiple Sclerosis and Neuromyelitis Optica. J Neurol Neurosurg Psychiatry (2011) 82(12):1355–9. doi: 10.1136/jnnp.2011.241836 21622936

[24] Peng F Zhong X Deng X Qiu W Wu A Long Y. Serum Uric Acid Levels and Neuromyelitis Optica. J Neurol (2010) 257(6):1021–6. doi: 10.1007/s00415-010-5455-1 20094725

[25] Lazzarino G Amorini AM Eikelenboom MJ Killestein J Belli A Di Pietro V. Cerebrospinal Fluid ATP Metabolites in Multiple Sclerosis. Mult Scler (2010) 16(5):549–54. doi: 10.1177/1352458510364196 20194579

[26] Liu C Xu Y Cui L Wang J Peng B Zhong L. Serum Uric Acid Levels and Their Correlation With Clinical and Cerebrospinal Fluid Parameters in Patients With Neuromyelitis Optica. J Clin Neurosci (2013) 20(2):278–80. doi: 10.1016/j.jocn.2012.02.042 23164828

[27] Niu PP Song B Wang X Xu YM. Serum Uric Acid Level and Multiple Sclerosis: A Mendelian Randomization Study. Front Genet (2020) 11:254. doi: 10.3389/fgene.2020.00254 32292418 PMC7133767

[28] Amorini AM Petzold A Tavazzi B Eikelenboom J Keir G Belli A. Increase of Uric Acid and Purine Compounds in Biological Fluids of Multiple Sclerosis Patients. Clin Biochem (2009) 42(10–11):1001–6. doi: 10.1016/j.clinbiochem.2009.03.020 19341721

[29] Shu Y Li H Zhang L Wang Y Long Y Li R. Elevated Cerebrospinal Fluid Uric Acid During Relapse of Neuromyelitis Optica Spectrum Disorders. Brain Behav (2017) 7(1):e00584. doi: 10.1002/brb3.584 28127508 PMC5256173

[30] Li X Yuan J Han J Hu W. Serum Levels of Homocysteine, Vitamin B12 and Folate in Patients With Multiple Sclerosis: An Updated Meta-Analysis. Int J Med Sci (2020) 17(6):751–61. doi: 10.7150/ijms.42058 PMC7085269 32218697

[31] Zhang J Li Y Zhou Y Zhao Y Xie H Duan R. Serum Homocysteine Level Is a Predictor of Relapse and Prognosis in Patients With First-Attack Neuromyelitis Optica Spectrum Disorders. Front Neurol (2021) 12:667651. doi: 10.3389/fneur.2021.667651 34122309 PMC8187771

[32] Ho PI Ortiz D Rogers E Shea TB. Multiple Aspects of Homocysteine Neurotoxicity: Glutamate Excitotoxicity, Kinase Hyperactivation and DNA Damage. J Neurosci Res (2002) 70(5):694–702. doi: 10.1002/jnr.10416 12424737

[33] Darendelioglu E Aykutoglu G Tartik M Baydas G. Turkish Propolis Protects Human Endothelial Cells *In Vitro* From Homocysteine-Induced Apoptosis. Acta Histochem (2016) 118(4):369–76. doi: 10.1016/j.acthis.2016.03.007 27085254

[34] Pan L Yin Y Chen J Ma Z Chen Y Deng X. Homocysteine, Vitamin B12, and Folate Levels in Patients With Multiple Sclerosis in Chinese Population: A Case-Control Study and Meta-Analysis. Mult Scler Relat Disord (2019) 36:101395. doi: 10.1016/j.msard.2019.101395 31521916

